# The Impact of Menstrual Cycle Phase on Economic Choice and Rationality

**DOI:** 10.1371/journal.pone.0144080

**Published:** 2016-01-29

**Authors:** Stephanie C. Lazzaro, Robb B. Rutledge, Daniel R. Burghart, Paul W. Glimcher

**Affiliations:** 1 Institute of Cognitive Neuroscience, University College London, London, United Kingdom; 2 Wellcome Trust Centre for Neuroimaging, University College London, London, United Kingdom; 3 Max Planck UCL Centre for Computational Psychiatry and Ageing Research, London, United Kingdom; 4 Department of Economics, Sacramento State University, Sacramento, California, United States of America; 5 Center for Neural Science, New York University, New York, New York, United States of America; Brock University, CANADA

## Abstract

It is well known that hormones affect both brain and behavior, but less is known about the extent to which hormones affect economic decision-making. Numerous studies demonstrate gender differences in attitudes to risk and loss in financial decision-making, often finding that women are more loss and risk averse than men. It is unclear what drives these effects and whether cyclically varying hormonal differences between men and women contribute to differences in economic preferences. We focus here on how economic rationality and preferences change as a function of menstrual cycle phase in women. We tested adherence to the Generalized Axiom of Revealed Preference (GARP), the standard test of economic rationality. If choices satisfy GARP then there exists a well-behaved utility function that the subject’s decisions maximize. We also examined whether risk attitudes and loss aversion change as a function of cycle phase. We found that, despite large fluctuations in hormone levels, women are as technically rational in their choice behavior as their male counterparts at *all* phases of the menstrual cycle. However, women are more likely to choose risky options that can lead to potential losses while ovulating; during ovulation women are less loss averse than men and therefore more economically rational than men in this regard. These findings may have market-level implications: ovulating women more effectively maximize expected value than do other groups.

## Introduction

Estrogen and progesterone levels have been shown to affect behaviors beyond those related to reproductive function (e.g. [1,2]). In fact, a variety of behavioral changes have been demonstrated with varying levels of these hormones: cyclic modulation of mood [3], changes in cognitive abilities [4], subjective responses to drugs such as cocaine [5] and amphetamines [6], ratings of trustworthiness [7], and willingness to compete in tournaments [8].

Functional neuroimaging studies have shown that brain activity varies across the menstrual cycle. Changes have been observed in reward- and arousal-related activity [9,10] and responses to emotional stimuli [11]. Estrogen and progesterone receptors are present in the nucleus accumbens [2,12,13], a key region in reward processing and decision-making. Since changes in brain activity related to reward and emotional processing are seen across the menstrual cycle, one might expect economic choice behavior to be affected by the changing levels of estrogen and progesterone across menstrual phase.

There is also evidence that hormone levels at the time of ovulation may influence decision-making in multiple domains. Numerous studies have examined the sexual and mating preferences of women during ovulation and it is well known that ovulation influences preferences for mates and male traits (e.g., [14]). Research has also shown that ovulation can affect consumer behavior such as clothing and food preferences [15,16]. In addition, gender differences between women and men exist in the economic domain. For example, women are more risk averse and less competitive than men on average [17]. While many studies have focused on the mate preferences of women around the time of ovulation, fewer have examined whether economic preferences change as a function of the menstrual cycle, and specifically whether risk aversion, loss aversion, and technical economic “rationality” change during ovulation. The lack of research on risk and loss preference changes during ovulation may relate to the difficulty in determining the time of ovulation and in testing during the narrow window that ovulation provides. We address this difficulty by tracking subjects over several months and using ovulation predictor kits to measure the surge in luteinizing hormone that leads to ovulation.

We thus first aimed to determine whether menstrual cycle phase is associated with alterations in technical economic rationality (the degree to which choices are internally consistent) in women. The core feature of rationality, and of particular interest to economists, is the notion of “transitivity”. A subject is said to be transitive and consistent if, for example, she demonstrates a preference for apples over oranges, oranges over pears ***and*** apples over pears. A subject is said to be irrational if she prefers apples over oranges, oranges over pears ***but also prefers*** pears over apples. Adherence to the Generalized Axiom of Revealed Preference (GARP) [18–20], a standard economic definition for rationality, is a necessary and sufficient condition for utility maximization. Such maximization requires transitive (i.e., non-circular) preferences. Moreover, GARP allows one to systematically search for very subtle violations of transitivity that are not as obvious as the example above. If women satisfy GARP, we can then examine what exactly it is that their behavior is maximizing. If they significantly fail to satisfy GARP at some particular cycle phase we can say meaningfully that their choice behavior is disorganized; that is, their choices cannot, in principle, be maximizing any possible subjective representation of value [19]. In contrast, a chooser who obeys GARP maximizes something (subjectively or objectively) and subsequent analysis can shed light on exactly what it is they are trying to maximize (consciously or not). If subjects adhere to GARP in all four tested phases of the menstrual cycle, this means that women behave as if they are maximizing some quantity, essentially a utility function of some kind, regardless of menstrual cycle phase. This critical first observation allows us to proceed to a second test–how does the object of maximization, the utility function, change across the menstrual cycle? To this end, we examined the risk preferences of women by presenting them with choices between safe options of certain monetary gains and risky options with an equal chance of either $0 or larger potential gains, a domain in which humans are typically rational, albeit risk averse [21]. Finally we examined whether there were changes across the menstrual cycle in *loss aversion*, a feature of human decision making whereby potential losses have a greater impact on choice than equivalent gains. We did this by presenting participants with choices between a safe option (a certain amount of $0) and a risky option offering equal chances of a gain and a loss. By determining the relative sizes of the gains and losses that are subjectively equivalent to a certain $0 outcome we measured the degree of loss aversion. Across all of these tests our goal was to characterize female economic choice behavior at four different phases of the menstrual cycle, including ovulation, compare it to male choice behavior, and see whether technical rationality or economic preferences differ between men and women at different phases of the menstrual cycle.

## Materials and Methods

### Subjects

39 female subjects were each tested four times during their natural menstrual cycle. No subjects were taking hormonal contraceptives or had been for the 4 months prior to the experiment and all subjects tracked their menstrual cycles for at least 3 months before beginning the experiment. Three subjects were excluded from all analyses for instruction-related issues and three subjects were excluded from the analysis of hormone levels for complications with one or more of the required blood draws. Behavioral data were analyzed for 36 subjects (mean age 24.75, range 18–38 years). Thirty-six male subjects (mean age 24.68, range 18–36 years) were each tested once on the same two tasks (the GARP experiment and the gambling experiment), but did not have their blood drawn. All subjects gave written informed consent to participate before each experimental session and the experimental paradigm was approved by the University Committee on Activities Involving Human Subjects at New York University.

### Experimental design

Female subjects were tested in four experimental sessions during their natural menstrual cycle, once during menses, once during the mid-follicular phase, once during ovulation and once during the luteal phase in a counter-balanced order across subjects. Each subject thus began the experiment in one phase and then proceeded in order through the remaining phases (e.g., nine subjects had the order 1) menses, 2) mid-follicular, 3) ovulation, 4) luteal, nine subjects had the order 1) mid-follicular, 2) ovulation, 3) luteal, 4) menses, etc.). Subjects tracked their menstrual cycles (start date and duration of menses) for at least 3 months prior to starting the experiment to ensure consistency in the number of days of each cycle and to determine cycle length (mean cycle length 29 days, range 23–39 days). Subjects who differed in cycle length by more than 3 days from cycle to cycle were not asked to participate in behavioral testing.

The exact timing of sessions was determined by the menstrual cycle of each subject. The menses test session was scheduled following self-report of menses onset (test session was on day 1–4 of the cycle). The mid-follicular test session occurred during the mid-follicular phase (after menses, but before ovulation: day 6–12 depending on cycle length). The ovulation test session was scheduled after detecting the luteinizing hormone (LH) surge that triggers ovulation. The LH surge was assessed with a commercially available at-home urine ovulation predictor kit (OvuQuick, Conception Technologies) that was given to participants on their first visit to the lab and the experimenter reminded them when to begin testing based on their shortest cycle length. Ovulation occurs approximately 24–36 hours following a positive result of an LH surge. Since the egg can live for up to 24 hours after being released, subjects came in for testing 24–48 hours after a positive result, at the time that they would have the greatest odds of conception. The luteal phase test session occurred after ovulation but before the next menses (at least 6 days after ovulation on day 20–31). Blood samples were acquired on each of the 4 test days to measure estradiol and progesterone serum levels. Subjects were not water or food deprived for the blood draws or experimental sessions and maintained normal eating and drinking habits. Male subjects were tested in one experimental session.

### Estradiol and progesterone assay and menstrual cycle phase monitoring

On each experimental day, one 4–6 mL blood sample was collected in a BD vacutainer serum tube (Fisher Scientific) by a registered nurse allowing measurement of serum estradiol and progesterone levels. After centrifugation, serum was collected and stored at -80 degrees Celsius until assayed. Blood samples were assayed by the New York University Core Clinical Lab. 17β-estradiol concentrations were determined using a commercially available enzyme immunoassay (Estradiol ELISA Kit; Cayman Chemical). Progesterone concentrations were determined using a commercially available enzyme immunoassay (Progesterone ELISA Kit; Cayman Chemical). Determination of serum estradiol levels confirmed that subjects (n = 33) tested during menses and mid-follicular phases had reduced levels of circulating estradiol (mean±sd, 151 ± 130 pg/ml during menses and 176 ± 151 pg/ml during mid-follicular) compared with ovulation (263 ± 244 pg/ml) and luteal phases (228 ± 205 pg/ml). Progesterone levels were also lowest during menses (2424 ± 1997 pg/ml) and mid-follicular phases (2533 ± 1990 pg/ml), higher during ovulation (3276 ± 2621 pg/ml) and highest during the luteal phase (6633 ± 5688 pg/ml), also confirming cycle phases. Each assay was run in duplicate and the results were averaged across the two samples.

### Experimental tasks

Stimuli were presented using E-Prime 1.2 (Psychology Software Tools Inc.) and involved an instructional practice session (approximately 5 minutes) immediately followed by the computerized decision-making portion. Choice situations in the experiments were presented in random order. At the conclusion of each experiment, one choice situation (one trial) was randomly selected to be realized for payment. At each session, female subjects received a show-up fee plus additional earnings based on the experiments as detailed below. Upon the completion of the fourth and final session, subjects also received a $50 bonus. Male subjects received a show-up fee plus additional earnings based on the experiments.

### GARP experiment

**Fig 1:**
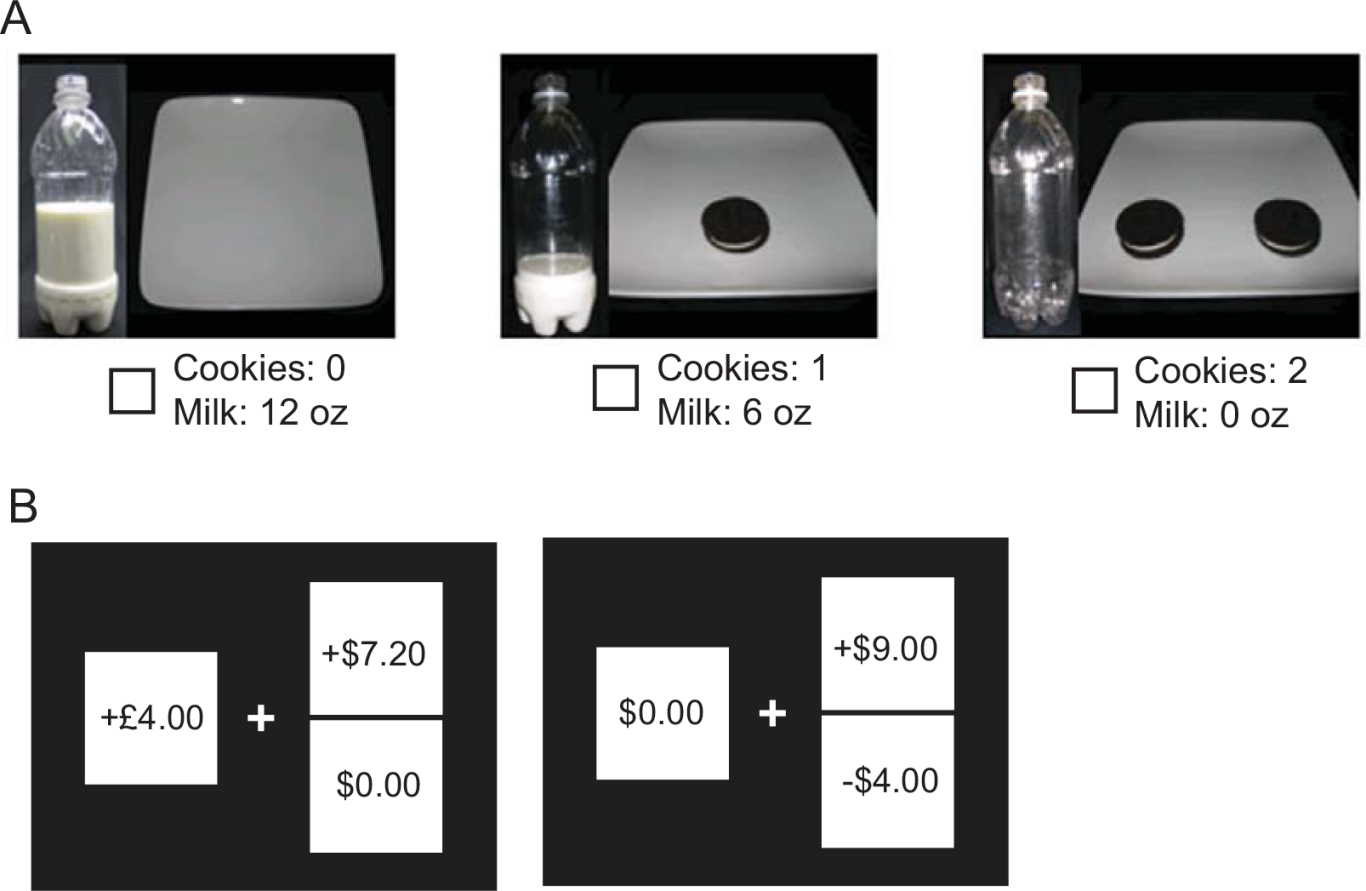
Example trials in the GARP and gambling experiments. **(A)** Example trial in the GARP experiment. This choice set offers three bundles. The first bundle contains no cookies and 12 ounces of milk. The second bundle contains one cookie and six ounces of milk. The third bundle contains no milk and two cookies. The subject indicates their choice by checking the appropriate box with a computer mouse. (**B)** An example *gain-only trial* (left) and an example *mixed gamble trial* (right) in the gambling experiment. Subjects indicated their choice by button press. See Methods for more details.

**Fig 2:**
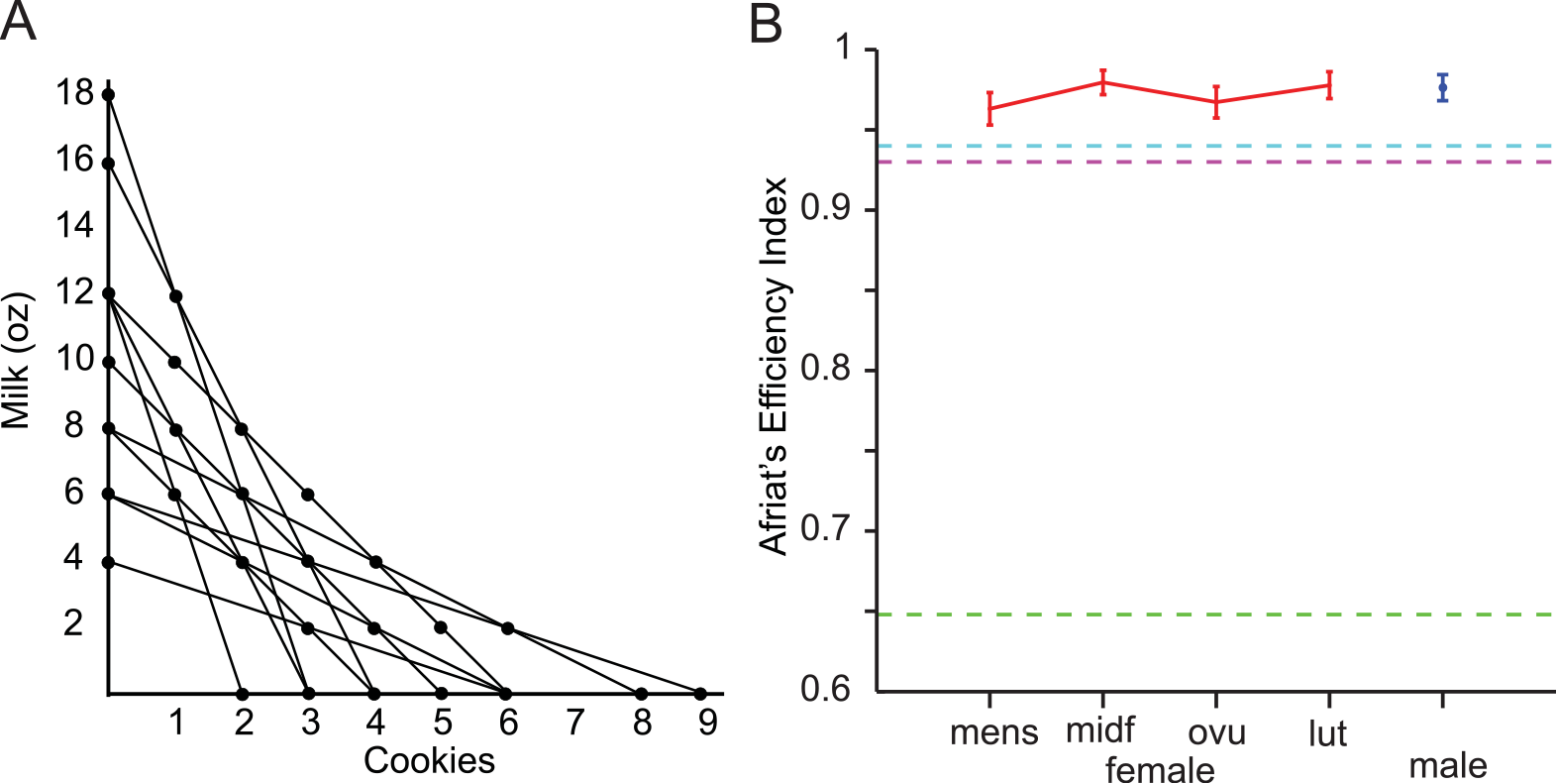
Results from the GARP experiment. **(A)** Budget sets used in the GARP experiment. Each line represents one budget set and circles on the line represent the bundles (or options) amongst which the subject can make a selection. (**B)** Rationality across the menstrual cycle. Measures of the Afriat’s Efficiency Index are plotted for subjects in each menstrual cycle phase and for an age-matched male control population (both n = 36 subjects). Measures for all phases were above 0.95, the common threshold for rationality, and not significantly differently across phases or compared to males (all p>0.3). Error bars represent standard errors. The dotted lines represent the AEI measurements for a random chooser (green) second grader (pink) and undergraduate (cyan) from Harbaugh et al. [22].

In the first experiment we tested adherence to GARP. The experimental design is similar to that designed by Harbaugh et al. [22] to examine whether children make technically rational choices about consumable goods. In our experiment, modeled precisely after the adult GARP experiment using food in Burghart et al. [23], which followed the Harbaugh et al. [22] design, subjects chose among bundles consisting of different numbers of cookies (zero to nine, in increments of one) and ounces of milk (zero to eighteen, in increments of two fluid ounces). Before testing began, subjects chose whether the task would involve Chips Ahoy Chocolate Chip Cookies (Nabisco) or Double Stuffed Oreos (Nabisco) and skim milk, 2% milk, whole milk, soy milk or water. Subjects then saw a total of 11 different choice sets (decision problems), presented in random order. Each choice set consisted of between three and seven bundles of cookies and milk that the subjects could choose amongst. **Fig 1A** shows an example choice set with 3 different bundles that the subject could choose from presented on a computer screen. Subjects were instructed to click inside of the checkbox under the bundle they wished to select. They could change their mind up until they clicked the “Next” button to move on to the next choice set. Subjects were instructed to simply choose the bundle from each screen that they wanted to receive. At the conclusion of the experiment, one choice set was randomly selected by the experimental software to be realized and the subject was served the number of cookies and amount of milk in their selected bundle. During the practice session, subjects saw an example of the computer randomly picking a choice set and indicating their chosen bundle. **Fig 2A** displays all 11 choice (budget) sets simultaneously as straight lines with filled circles representing the bundles included in each choice set. We note that the specific test of GARP we employed also tests what is called “weak monotonicity;” that increasing quantities of a preferred good are themselves preferred. There is no doubt that for many goods when subjects possess a sufficient quantity of the good, additional quantities may be aversive. We intentionally designed our choice sets to minimize the possibility that our subjects had weakly monotonic preferences—which they did. Consistent, technically rational, choice behavior that violates weak monotonicity would have called for a more complex analysis than presented here.

### Gambling experiment

Subjects were endowed with $20 and told that with this money they would be gambling. They were instructed that the most they could lose was the $20 they had just been given and that they could win up to $20 more based on their choices. Subjects made a total of 100 choices. Each trial offered a choice between a certain amount of money (ranging in value from $0 to $8) and a gamble with equal probabilities of either of two possible outcomes.

#### Risk-Aversion

Subjects faced 26 different choice situations of the 100 (the *gain-only trials*) that consisted of choosing between a safe option, a strictly positive amount ranging from $2 to $8 and a risky option that offered a 50% chance of winning a positive amount of money ($2.40 to $19) and a 50% chance of winning $0. We used these gain-only gamble choices to estimate the degree of risk aversion. **Fig 1B (left)** shows an example of a gain-only gamble trial.

#### Loss-Aversion

Subjects also faced 74 choice situations in which they were given a choice between a certain amount of $0 (they would neither lose nor gain anything) and a risky option that offered a 50% chance of winning some positive amount of money ($2 to $10) and a 50% chance of losing some amount of money ranging from $0.10 to $18, (so-called *mixed gamble* trials). We used these mixed gamble trials to estimate each subjects degree of loss aversion. **Fig 1B (right)** shows an example of a mixed gamble trial. The gain-only and mixed gamble trials were randomly inter-leaved. After the subject completed 100 choice trials, one trial was randomly chosen by the subject for payment by rolling a 100-sided die. If the choice in the randomly selected trial was the certain monetary amount, the subject was given that amount of money. If the choice was the gamble, it was resolved by an actual coin toss called by the subject. Subjects were informed at the start of the experiment that only one choice would be randomly selected for payment at the end of the experiment.

## Results

### Subject hormonal state

**Fig 3:**
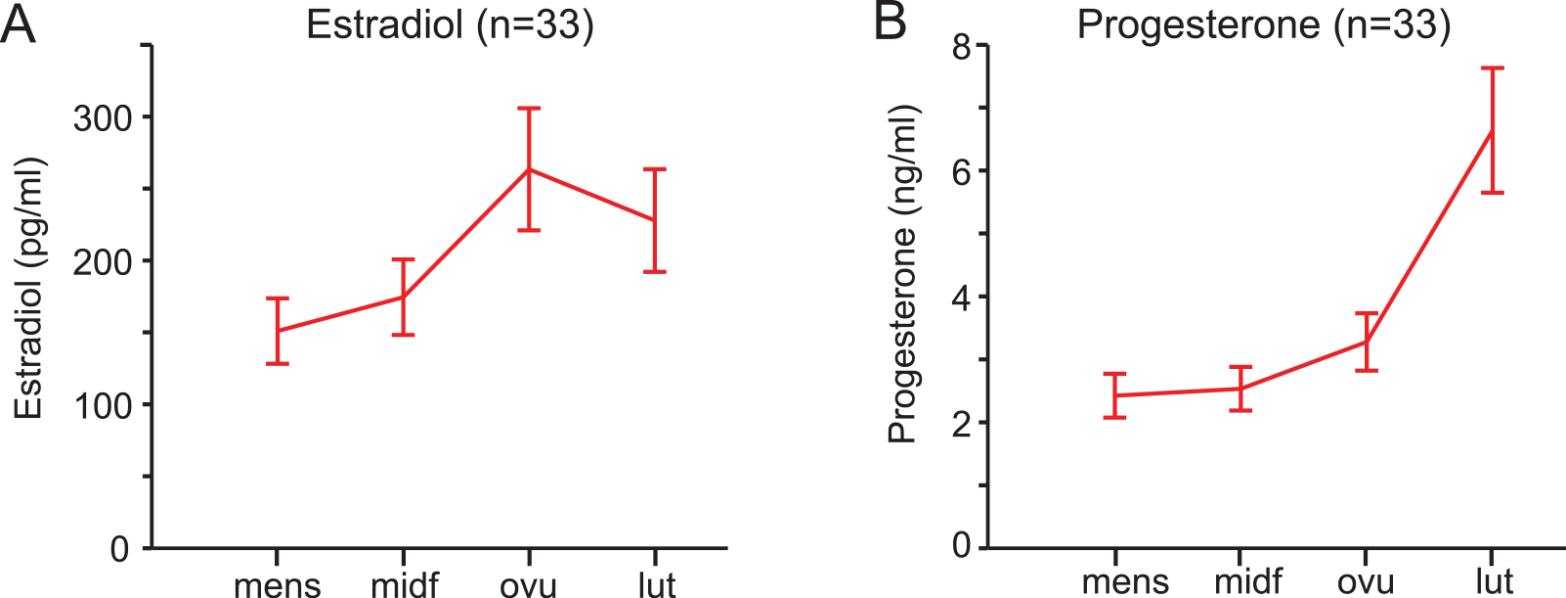
Hormone levels in the subject population. **(A)** Average estradiol levels in 33 subjects, all with blood samples in all four phases. Differences between adjacent phases are significant at p<0.01 except between ovulation and the luteal phase (p = 0.063). **(B)** Average progesterone levels. All differences between adjacent phases are significant at p<0.01 except between menses and the mid-follicular phase (p = 0.52) and between the mid-follicular phase and ovulation (p = 0.077).

During a normal menstrual cycle, women exhibit natural variations in levels of the ovarian hormones estrogen and progesterone [24]. We tested subjects during four phases of their natural menstrual cycle: menses (early-follicular), mid-follicular, ovulation and luteal (see Methods) and measured serum estrogen and progesterone levels to confirm cycle phase. Average serum levels of estradiol and progesterone levels in our subjects follow the normal pattern (**Fig 3**). Average serum levels of estradiol (**Fig 3A**) were significantly higher in the mid-follicular phase than during menses (paired t-test, p<0.01). Additionally, estradiol was significantly higher during ovulation and the luteal phase than menses and mid-follicular (paired t-tests, all p<0.05). Progesterone levels (**Fig 3B**) were lowest during menses and the mid-follicular phase, and these levels were not significantly different from one another (p = 0.52). The luteal phase had the highest level of serum progesterone and levels were significantly higher than in all other phases (all p<0.01). These results confirm that our population showed the typical hormonal level patterns during the menstrual cycle for young adult women [25].

### Testing rationality

Our first goal was to establish if menstrual cycle phase alters the *technical rationality* (the degree to which choices are internally consistent) of women. Subjects who adhere to GARP can be described as fully transitive and logically consistent in their choice behavior. Put another way, their choices are consistent with the idea that they are trying to maximize a self-defined goal. A consistent and transitive chooser follows this logic in their choice behavior: if they choose A over B and B over C, they must also choose A over C. Adherence to GARP is a mainstay of economic analysis because subjects who violate GARP from moment to moment cannot, in principle, be maximizing towards any single or stable goal with their choice behavior [19] and are hence said to be *irrational*. In contrast, a chooser who obeys GARP can be described as pursuing a stable maximization goal of some kind, and subsequent analysis can then determine exactly what it is they are trying to maximize (consciously or not).

In our GARP experiment, subjects chose between economic ‘bundles’ of goods, combinations of a certain number of cookies and a certain amount of milk (**Fig 1A**). Following a well-established procedure [22,23,26], 11 budget (choice) sets (**Fig 2A**), each consisting of between three and seven alternative combinations of milk and cookies (bundles) were presented as real offers to our subjects. Their task was simply to say which they preferred. Choice sets among bundles of milk and cookies can be thought of as technical economic ‘budget sets’ where cookies and ounces of milk are offered at a fixed price per cookie and a fixed price per ounce of milk and the chooser has a fixed amount of money to spend on these goods. For example, the choice set in **Fig 1A** shows a choice set consisting of three bundles. It shows a bundle with 0 cookies and 12 ounces of milk, a bundle with 1 cookie and 6 ounces of milk, and a bundle with 2 cookies and 0 ounces of milk. This ‘budget set’ thus offers each cookie at three times the cost of 2 ounces of milk because the subject can only ‘afford’ 2 cookies or 12 oz (6 units) of milk. Bundles in each budget set fall on a line in two-dimensional space defined by the numbers of cookies and ounces of milk. The full set of 11 budget sets can be seen in **Fig 2A**. The slope of the line depends on the ratio of the price of cookies to the price of two ounces of milk. A steeper slope reflects more expensive cookies and a shallower slope reflects more expensive milk. A violation of GARP can be seen in the following examples: a subject selects bundle A over bundle B, and then selects bundle B over bundle C that has the same or more of each good as in bundle A. For the purposes of this study we explicitly assume that over the range of rewards we examine choosers can never face a situation in which an increase in the quantity of milk or number of cookies is considered aversive. Allowing for that possibility would significantly complicate our analysis of consistency.

We then determined whether or not the choices made during each tested menstrual cycle phase were internally consistent in their structure (adhered to GARP), and evaluated the severity of any violations observed. It is important to note that consistency and preference are *not* entangled in this procedure. A subject can be perfectly consistent and highly risk averse, could prefer milk over cookies or the reverse. Consistency is not about what the subject prefers, or how cookies and milk trade-off against one another, but rather is about whether there exists some logical rationale that could explain their choices.

We employed Afriat’s Efficiency Index (AEI) [20] as a metric for rationality. An AEI of 1 indicates perfect consistency and identifies a chooser who is perfectly transitive. An AEI above 0.95, a chooser whose total set of gains and losses is better than 95% of what could be achieved with perfectly transitive choices, is considered to be above the common threshold for technical rationality [27]. Like other experiments in the literature, our choice sets were designed to elicit single subject measures of rationality ranging from 0.85 to just below 1 –a calibration intended to minimize ceiling and floor effects in our measurements.

The degree of rationality observed in our female subjects is similar both to the males we tested and to that observed in other studies. The AEI mean ± s.e.m. for our subjects (n = 36) during menses was 0.963 ± 0.010, mid-follicular 0.980 ± 0.008, ovulation 0.967 ± 0.010, and luteal 0.978 ± 0.008. The average AEI for our male subjects (n = 36) was 0.976 ± 0.008. Women in all phases of the menstrual cycle had an average AEI above 0.95, indicating that our subjects remain consistent in their choice behavior and are not only technically rational across the menstrual cycle but are as rational as their male counterparts at all times (all comparisons, p>0.1, **Fig 2B**).

There was thus no evidence in our dataset that the degree of technical rationality of ovulating human females fluctuates during the menstrual cycle. Their behavior was always logically consistent; they maximized a utility function of some kind regardless of menstrual cycle phase. This critical first observation allows us to proceed to a second test: Do their logically describable preferences change during the monthly cycle? Put more technically, does the utility function being maximized change across the menstrual cycle?

### Measuring risk tolerance

To measure risk aversion (the precise degree of utility function curvature), in a second ‘gambling’ experiment we offered subjects choices between certain amounts of money and ‘gain-only’ gambles with equal probabilities of winning a larger amount of money or winning $0 (**Fig 1B, left**). We systematically varied the certain amounts and how much the subjects stood to win in the gambles to allow us to estimate the degree of risk aversion in our subjects, and to determine whether risk attitudes were affected by menstrual cycle phase.

**Fig 4:**
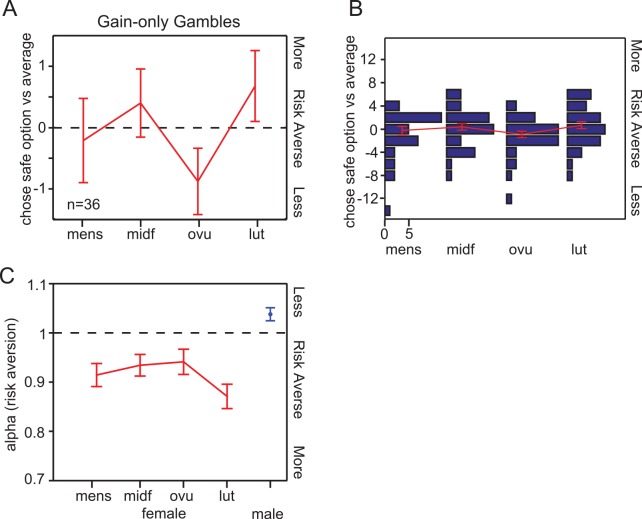
Results for gain-only trials in gambling experiment. **(A)** Effect of menstrual phase on the number of times subject chose the safe/certain option over the risky option in gain-only trials. (**B)** Histogram of the number of times the safe option was chosen by each individual subject, computed as the difference between the number of safe choices in each cycle phase and the average number of safe choices across cycle phases. **Fig 4A** is superimposed on the histogram for comparison. (**C)** Effect of menstrual phase on risk aversion. Parameter fits are estimated simultaneously for all phases with controlling for session order effects (see **S1 File** for details on the fitting procedure). Parameter estimates for an age-matched male population are also plotted on this for comparison. Subjects were more risk averse during the luteal phase than during ovulation (p = 0.048) or the mid-follicular phase (p = 0.056).

To test for changes in risk preferences across the menstrual cycle, we considered the number of times each subject chose the safe option over the risky gamble in each phase relative to the number of safe option choices across phases in each individual (**see Fig 4A and 4B**). We did identify a marginally, or nearly, significant (two-tailed Wilcoxon sign rank, p = 0.062) reduction in risk aversion in the ovulation phase compared to the luteal phase. No other significant effects on risk aversion by phase were observed (ANOVA, p = 0.26). Although a larger study with the same four-month tracking of subjects procedure that we employed would be very expensive, such a study might reveal a more robust increase in risk-tolerance during ovulation, a finding that might be of evolutionary-reproductive relevance. We also note that using a one-tailed statistical test would have rendered this result significant at the standard 0.05 threshold.

To better understand the stationarity of risk attitudes over the menstrual cycle and to allow comparison with parametric measures in the literature, we also used a simple model-based approach to estimate the curvature of the utility function (the quantitative measure of risk attitude) for each subject. We assumed a power utility function and fit a risk aversion coefficient, alpha, using standard model-fitting procedures [28,29].

With a simple power utility model of this type a risk aversion coefficient of 1 corresponds to risk-neutral choice behavior and thus describes a subject who maximizes expected value on average. A risk aversion coefficient greater than 1 yields an expansive utility function and indicates risk-seeking behavior, while a coefficient less than 1 indicates the typical compressive utility function of risk-averse choice behavior.

**Fig 5:**
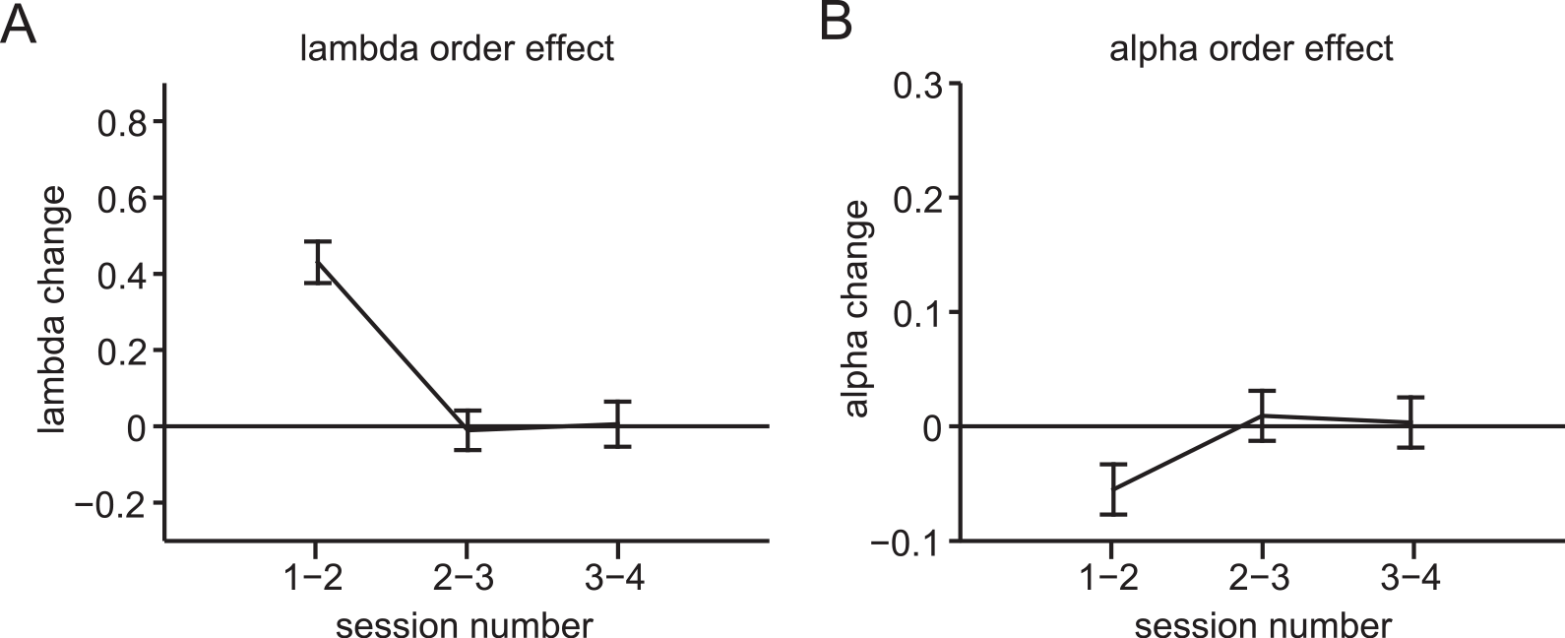
Session order effects. **(A)** Session order effects on loss aversion. Loss aversion increased from the first to second session, but remained stable for all subsequent sessions. Note that session order was randomized with regard to cycle phase. (**B)** Session order effects on risk aversion. Risk aversion increased from the first to second session, but remained stable for subsequent sessions.

After controlling for session order effects (see Methods and **Fig 5B**), the risk aversion coefficient that best accounted for average choice behavior (mean ± s.e.) during menses was 0.914 ± 0.024, mid-follicular was 0.934 ± 0.022, ovulation was 0.941 ± 0.026, and luteal was 0.870 ± 0.025 (all n = 36). We found that on average, our subjects were risk-averse at all phases of the menstrual cycle (all p<0.05), and were more risk averse than males (p<0.05, **Fig 4C**), as has been previously noted [17,23,30]. We found that the degree of risk aversion did not significantly vary across the menstrual cycle, but there was once again an interesting tendency for our subjects to be less risk averse during ovulation than the luteal phase (p = 0.048; **Fig 4C**). It is worth noting that any degree of risk aversion can be characterized as rational, so long as the subjects remain internally consistent in their choice preferences; this finding does not run counter to our observation that our subjects were consistent in their choices.

### Assessing loss aversion

Even in largely transitive and rational choosers, however, there is compelling evidence that some classes of situations (for example, situations which include monetary losses) robustly induce technically irrational choice behavior. These are situations where classical maximization theory widely fails. In 1979, Kahneman and Tversky documented one of the most profound of these phenomena, reporting that potential monetary losses have a greater impact on decisions than equivalent monetary gains, a property referred to as *loss aversion* [31]. Loss aversion is, for most technical theorists, an irrational feature of human decision-making because it leads choosers to make sets of decisions that are logically circular and violate GARP. Loss aversion has been widely studied and demonstrated consistently in both men and women [28,32–34]. To assess whether attitudes towards potential monetary losses (and thus the degree of this type of irrationality) varied across the menstrual cycle in women, in some trials of our ‘gambling’ experiment we offered subjects choices between a certain outcome of gaining and losing nothing (an amount of $0) and a gamble that offered equal probabilities of winning one amount of money or losing another amount of money (**Fig 1B, right**). A typical loss-averse subject prefers a certain outcome of $0 to a 50% change of winning $18 and a 50% chance of losing only $10. To assess the degree of loss aversion in our subjects, we systematically varied the gamble win and loss amounts and asked our subjects to choose amongst these options during each menstrual cycle phase.

**Fig 6:**
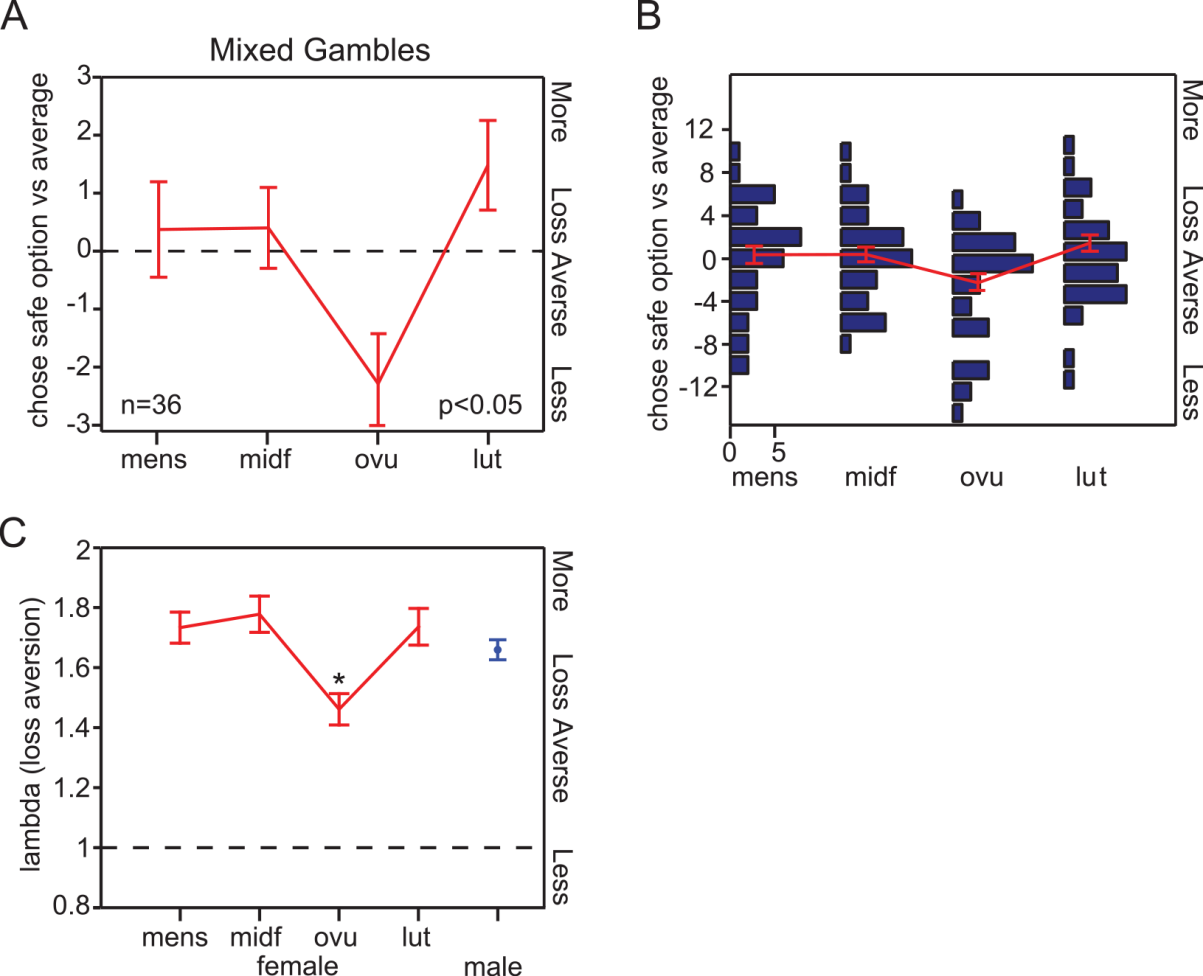
Results from the gambling experiment for loss aversion. **(A)** Effect of menstrual cycle phase on the number of times the safe certain option ($0) was chosen over the *mixed gamble* (ANOVA, p<0.01). **(B)** Histogram of the number of safe options chosen by each individual subject, computed as the difference for each subject between the number of safe options in each cycle phase and the average number of safe options chosen across cycle phases. Fig 6A is superimposed on the histogram to display group average and s.e.m. **(C)** Effect of menstrual cycle phase on loss aversion. Parameter fits are estimated simultaneously for all phases with the single best noise parameter (0.94 ± 0.04) controlling for session order effects. Parameter estimates for an age-matched male population are also plotted. Subjects are less loss averse during ovulation than other phases (all p<0.001). Loss aversion in menses, mid-follicular and luteal phases did not differ significantly from each other (all p>0.3).

To quantify the degree of loss aversion in our subjects, we considered the number of times each subject chose the safe option over the risky option in each phase relative to the average number of safe option choices across phases in each individual (**Fig 6A and Fig 6B**). Here we found a significant effect of phase on the number of safe options chosen (ANOVA, p<0.01). Specifically, women chose significantly more gambles and were *less loss averse* during ovulation than the luteal phase (two-tailed Wilcoxon sign rank, p<0.01).

To better understand the non-stationarity of loss aversion over the menstrual cycle and to allow comparison with other parametric measurements in the literature, we used a standard model-based approach [28] to estimate the average degree of loss aversion in each menstrual cycle phase. When using this procedure, a loss aversion coefficient, lambda, equal to 1 indicates gain-loss neutrality. This describes a subject who treats the loss of a dollar as equal and opposite to the gain of a dollar in terms of the choices they make. A lambda greater than 1 captures loss-averse choice behavior, a subject who treats the loss of a dollar as being more serious than the gain of a dollar. For example, a lambda of 2 indicates that the loss of $1 would loom as large to that subject as a gain of $2. Reports in the literature indicate that loss aversion coefficients of around 2 are typical of human choosers with women typically being more loss averse (having higher lambdas) than men [34].

After controlling for session order effects (see Methods and **Fig 5A**), the loss aversion coefficient, lambda, that best accounted for subject choice behavior during menses was 1.733 ± 0.052 (mean ± s.e.), mid-follicular 1.778 ± 0.061, ovulation 1.460 ± 0.52, and luteal 1.736 ± 0.061, (all n = 36). These parameter estimates indicate that on average, our subjects were significantly loss averse in all 4 tested phases of the menstrual cycle (all p<0.001). However, we found that our subjects were significantly less loss averse during ovulation than during all other phases (all comparisons, p<0.001; **Fig 6C**). Loss aversion in menses, mid-follicular and luteal phases did not differ significantly from each other (all p>0.3). Interestingly, the loss aversion coefficient estimated for our male subjects was also not significantly different from that of our female population during menses (p = 0.22), mid-follicular (p = 0.081), or luteal phases (p = 0.26). We found that our male subjects were significantly more loss averse than the ovulating women in our study (p = 0.001). These data indicate that loss aversion is modulated by menstrual cycle phase. During ovulation, women are significantly less loss averse than men. To some theorists, this means that women, during ovulation, are more technically rational than men.

## Discussion

Women are most fertile when they are ovulating and are only fertile in the few days around ovulation (approximately 10% of the days during each cycle) [35,36]. The Ovulatory Shift Hypothesis [37,38] proposes that natural selection may have influenced a shift in certain aspects of female psychology during this brief window when conception is possible. Much of the research examining the Ovulatory Shift Hypothesis has been in relation to mate choices as mate choice has significantly higher consequences when women are ovulating. Indeed, during fertile days in their cycles, women show a shift in their preferences for male traits such as preferring more masculine faces [39,40] and deeper male voices ([41]; although see [42] for a critique of these findings). Consumer product choice behavior also shifts, with women choosing ‘sexier’ clothing and fashion accessories near peak fertility [15] and seeking positional goods near ovulation that improve their social standing [43]. One possible explanation for this behavior is that self-esteem decreases at this point in the ovulatory cycle, which translates into an increased willingness to spend money to augment attractiveness during the time of high fertility [44]. This increased willingness to spend money during ovulation may relate to our finding that women become more tolerant of potential financial losses during ovulation.

In competitive bidding tasks, when women have the opportunity to compete with other women, they are less competitive during the luteal phase than other phases [45]. This has been interpreted as competitiveness being less desirable during this infertile phase of the menstrual cycle. Interestingly, we also find a marginally significant tendency for women to be more risk averse during the luteal phase than other phases. Women bid as if they are more risk averse than men in all phases except during ovulation [46]. Women may be more likely to take risks during this high fertility phase in order to increase the probability of conception. Furthermore, women give less than men in a trust game except around the time when ovulation is likely to occur and also offer more than men in an ultimatum bargaining game only during this time [45]. These increases in financial giving when ovulation is more likely to occur may reflect an increased willingness to tolerate financial losses, lending support for our novel finding that women become less loss averse when ovulating.

In recent years, a small but increasing literature has begun to examine the relationship between hormones and economic behavior. Some papers published in this literature have begun to use menstrual cycle phase as a proxy for the effect of ovarian hormones on economic behavior (e.g., [46,47]). Although this is a new and growing area of inquiry, it already seems clear that understanding how the menstrual cycle influences choice behavior could account for a number of important economic factors that have so far gone largely unstudied and hence been largely unexplained [48]. As Pearson and Schipper [46] have noted, “robust findings on the endocrinology of economic behavior could profoundly influence our understanding of the biological basis of economic outcomes including the gender wage gap.” This is particularly interesting because while the general public often assumes that fluctuating hormones during the menstrual cycle affect the rationality of women, experimental and theoretical economists have widely assumed the opposite; that cycle phase is irrelevant for economic behavior. Our data identify a third position. Cycle phase does matter in choice behavior. Women express different choice behavior at different times–although never in an irrational way. This is a critical observation that economists have overlooked and which could have real impact in our understanding of economic behavior. One might, of course, be tempted to respond to this observation by arguing that as long as the female decision-makers in real world choice situations equally represent all possible cycle phases then accounting for phase will be unnecessary. We note that this will be true *only* when the female decision-makers in a group cannot self-select; when they cannot choose whether or not to participate in the market or purchase situation of interest. This may not be case in most real-world situations; we simply do not know. Intuitively sophisticated female decision-makers may defer choices of particular kinds to particular times. If this is the case, then comparing choice behavior across two or more kinds of decisions may be important for modeling self-selection by cycle phase. Paradoxically, it may well be the case that accounting for cycle in economic models could rationalize choice behavior in real populations.

### Conclusions

We found that ovulating human females are technically rational (internally consistent) at all phases of the menstrual cycle. In fact, we found that there is almost no change in the degree of rationality over the course of the cycle and the degree of rationality is not different than that of a matched population of male subjects. Women’s economic preferences do, however, change across the menstrual cycle. At the beginning of the cycle, during the follicular phase, and at the end of the cycle, during the luteal phase, women are as loss averse as their male counterparts. When ovulating, however, women are less loss-averse than males, meaning they are more willing to tolerate potential economic losses, and behave more similarly to an agent that maximizes the expected value of its choices. This result may have important implications for market behavior. Our data also demonstrate that tests of economic behavior made on female subjects, especially those related to loss aversion, could account for the menstrual cycle phase of study participants. This may be of particular importance in interpreting existing results if subjects participating in behavioral studies self-select for cycle phase when volunteering for economic experiments.

## Supporting Information

S1 Data Experimental data are included for females for all menstrual cycle phases and for males. (ZIP)

S1 File Experiment methods are detailed for GARP experiment analysis and gambling experiment analysis. Model fitting details are included.
(DOCX)
