# Supplementary material for: The Impact of Menstrual Cycle Phase on Economic Choice and Rationality
Source: PLoS One. 2016 Jan 29;11(1):e0144080. doi: 10.1371/journal.pone.0144080 (PMC4732761; doi:10.1371/journal.pone.0144080)
Supplement: S1 File — Model fitting details are included. (DOCX) [file pone.0144080.s002.docx]

**Supporting Information**

**GARP experiment analysis.** The severity of GARP violations was measured using Afriat’s Efficiency Index [22,27]. Choice sets among bundles of milk and cookies can be thought of as “budget sets” where cookies and ounces of milk are offered at a fixed price per cookie and a fixed price per ounce of milk with the chooser having a fixed amount of money to spend on these goods. For example, the choice set in **Fig. 1A** describes a decision in which the subject must choose between three options, or ‘bundles’. The first bundle offers 0 cookies and 12 ounces of milk, the second offers 1 cookie and 6 ounces of milk, and the third bundle offers 2 cookies and 0 ounces of milk. This ‘budget set’ thus offers each cookie at three times the cost of 2 ounces of milk. (The subject can only afford a maximum of 2 cookies or 12 oz (6 units) of milk.) Bundles in each budget set thus fall, in this completely standard approach from consumer theory, on a line in two-dimensional space defined by the numbers of cookies and ounces of milk. The full group of 11 budget sets (and hence 11 choice situations) can be seen in **Fig. 2A**, and is modeled on the choice sets used in Harbaugh et al. [22] and Burghart et al. [23].

**Gambling experiment and model-based analysis.** We used a standard 3-parameter model (α, λ, and µ) to estimate risk and loss aversion in our subjects [28] as an extension and confirmation of our non-parametric analysis. The subjective values of potential gains and losses were estimated using the following two equations where x is the monetary amount, α reflects the curvature of the subjective value function and λ is the loss aversion coefficient:

$$If x\geq0, v\left( x \right)=x^{\alpha}$$

$$If x<0, v\left( x \right)=-\lambda\left( -x \right)^{\alpha}$$

We used the softmax equation to estimate the probability of a gamble being accepted by comparing the difference between the subjective values of the gamble and the certain amount of money offered to the subject, where µ is the noise parameter:

$$P\left( gamble acceptance \right)=\frac{1}{1+e^{-\mu\left( v\left( gamble \right)-v\left( certain \right) \right)}}$$

When α = 1, this indicates risk neutrality. When α > 1, this indicates risk seeking behavior, and when α < 1, this indicates risk aversion. When λ = 1, this indicates gain-loss neutrality, that equivalent gains and losses have a similar impact of choice. When λ > 1, subjects are loss averse and when λ < 1, subjects overvalue gains relative to losses. We estimate parameters by the method of maximum likelihood for each of our four menstrual cycle phase populations, constraining µ to be the same across populations. For both α and λ, we also included parameters for session number to control for session order effects. For male subjects, we estimate both α and λ, constraining the noise parameter to be the same as in the female population. Confidence intervals on parameter estimates were computed from the inverse of the Hessian matrix. We compared these maximum-likelihood parameter estimates by Wald test. In addition to our model-based approach, we also tested for differences in choices between phases by comparing the number of gambles chosen in each menstrual cycle test phase using Wilcoxon sign-rank tests.
